# Assessment of the cost-effectiveness of Australia's risk-sharing agreement for direct-acting antiviral treatments for hepatitis C: a modelling study

**DOI:** 10.1016/j.lanwpc.2021.100316

**Published:** 2021-11-23

**Authors:** Dr Nick Scott, Ms Anna Palmer, Mr Tom Tidhar, Prof Mark Stoove, Dr Rachel S. Sacks-Davis, A/Prof Joseph S. Doyle, Dr Alisa J. Pedrana, Prof Alexander Thompson, Prof David P. Wilson, Prof Margaret Hellard

**Affiliations:** a Disease Elimination Program, Burnet Institute, Melbourne, VIC 3004, Australia; b Department of Epidemiology and Preventive Medicine, Monash University, Melbourne, VIC, 3004, Australia; c Department of Infectious Diseases, The Alfred and Monash University, Melbourne, VIC 3004, Australia; d Department of Medicine, The University of Melbourne, Parkville, VIC 3050, Australia; e Department of Gastroenterology, St Vincent's Hospital Melbourne, Melbourne, VIC 3165, Australia; f Peter Doherty Institute for Infection and Immunity, Parkville, Australia; g School of Population and Global Health, University of Melbourne

**Keywords:** cost-effectiveness, elimination, hepatitis C, mathematical model, productivity, DAA, direct-acting antiviral, GDP, gross domestic product, PWID, people who inject drugs, QALY, quality-adjusted life year, WHO, World Health Organization

## Abstract

**Background:**

Hepatitis C elimination may be possible with broad uptake of direct-acting antiviral treatments (DAAs). In 2016 the Australian government committed A$1.2 billion for five years of unlimited DAAs (March 2016 to February 2021) in a risk-sharing agreement with pharmaceutical companies. We assess the impact, cost-effectiveness and net economic benefits likely to be realised from this investment.

**Methods:**

Mathematical modelling to project outcomes for 2016-2030 included: (S1) a counter-factual scenario (testing/treatment maintained at pre-2016 levels); (S2) the current status-quo (testing/treatment as actually occurred 2016-2019, with trends maintained to 2030); and (S3) elimination scenario (S2 plus testing/treatment rates increased between 2021-2030 to achieve the WHO elimination targets).

**Findings:**

S1 resulted in 68,800 new hepatitis C infections and 18,540 hepatitis C-related deaths over 2016-2030. The total health system cost (HCV testing, treatment, disease management) was A$3.01 billion and the cost of lost productivity due to absenteeism, presenteeism and premature deaths was A$26.14 billion. S2 averted 15,700 (23%) new infections and 8,500 (46%) deaths by 2030, with a total health system cost of A$3.48 billion, A$472 million more than S1 (A$1.65 billion more in testing/treatment but A$1.20 billion less in disease costs; A$5,752 per QALY gained from a health systems perspective). Productivity loss over 2016-2030 was A$19.96 billion, A$6.17 less than S1, making S2 cost-saving from a societal perspective by 2022 with a net economic benefit of A$5.70 billion by 2030. S3 averted an additional 10,000 infections and 930 deaths compared with S2 and increased the longer-term economic benefit.

**Interpretation:**

Five years of unrestricted access to DAAs in Australia has led to significant health benefits and is likely to become cost-saving from a societal perspective by 2022.

**Funding:**

Burnet Institute

**Research in context**

**Evidence before this study**

The global scale up of direct-acting antiviral treatments (DAAs) for hepatitis C has been limited by their cost and affordability. For countries considering investing in hepatitis C treatment scale-up, modelling studies and investment cases are available to estimate what might be good value for the price of DAAs. However, these estimates do not necessarily align with program implementations due to unknown real-world constraints. Evidence is needed on the real-world cost, impact and cost-effectiveness of hepatitis C elimination programs to inform whether they can actually achieve their estimated impact and return on investment.

**Added value of this study**

Australia is in a fortunate position globally with regards to hepatitis C elimination. Through a volume‐based, risk‐sharing agreement with originator pharmaceutical companies, the Australian government committed A$1.2 billion for unlimited DAA treatment courses between March 2016 and February 2021. This arrangement means that Australia has the policy environment and upfront investment to achieve hepatitis C elimination. Now that this five-year scale-up period has expired, it is possible to assess the actual impact and cost-effectiveness that was achieved by Australia's national strategy to finance treatments.

**Implications of all the available evidence**

We found that compared to a scenario with no scale-up, under current testing/treatment numbers, Australia is on track to have averted 15,700 (23%) infections and 8,500 (46%) deaths over 2016-2030. When savings from disease cost averted are considered, the additional health system costs over 2016-2030 are estimated to be A$472 million more than without DAA scale-up (A$5,752 per QALY gained from a health systems perspective). Importantly, economic productivity gains from hepatitis C cure are estimated to be A$6.17 over 2016-2030, making Australia's investment strategy on track to become cost-saving from a societal perspective by 2022 with a net economic benefit of A$5.70 billion by 2030.

In addition, despite the favourable policy environment we also found that more work is needed to achieve elimination. As well as informing the Australian government's ongoing elimination efforts, this work can support other countries in their own price negotiations by setting realistic expectations for what hepatitis C elimination programs can achieve.

Alt-text: Unlabelled box

## Introduction

The discovery of direct-acting antiviral treatments (DAAs) to cure hepatitis C is one of the most significant biomedical advances of the last decade and has made elimination of hepatitis C as a public health threat a realistic goal.^1^^,^^2^ With >95% cure,^3^, ^4^, ^5^ DAAs have the potential to substantially reduce hepatitis C morbidity, mortality and transmission.^6^, ^7^, ^8^ In response the World Health Organization (WHO) set 2030 elimination targets of an 80% reduction in hepatitis C incidence and a 65% reduction in hepatitis C-related mortality compared to 2015 levels.^1^ Despite this, DAAs remain unavailable or available with restricted access in a majority of countries.

The global scale up of DAA treatment has been limited by many factors, including a lack of enabling laws, policies and guidelines to support elimination programs, limited awareness and advocacy to drive demand, and limited infrastructure and skilled workforces to support program implementation.^7^^,^^8^ Additionally, a major barrier is the cost and affordability of both tests and treatments. Modelling studies and investment cases are available to guide what might be good value for the price of DAAs and diagnostics but model projections do not necessarily align with program implementations due to unknown real-world constraints. Evidence is needed on the real-world cost, impact and cost-effectiveness of hepatitis C elimination programs.

Australia is in a fortunate position globally with regards to hepatitis C elimination. Through a volume‐based, risk‐sharing agreement with originator pharmaceutical companies, the Australian government committed around A$1.2 billion for unlimited DAA treatment courses between March 2016 and February 2021^9^. With no cap on treatment numbers, a public health response was incentivised and DAAs were made available to all Australians regardless of disease stage or reinfection risk.^10^Between March 2016 and December 2019, an estimated 82,280 treatment courses were initiated in Australia,^11^ representing approximately 45% of the estimated 189,000 people who were living with hepatitis C in 2015.^12^ This arrangement means that Australia has much of the policy environment to achieve hepatitis C elimination, albeit with ongoing challenges around service access, diagnosis and retention in care^13^, ^14^, ^15^ of people with hepatitis C.

In this paper we use mathematical modelling to assess the impact, cost-effectiveness and net economic benefits that are likely to be realised from Australia's investment in hepatitis C treatment. We also assess the potential impact, cost-effectiveness and net economic benefits of enhancing efforts to achieve the 2030 WHO elimination targets. As well as informing the Australian government's ongoing elimination efforts, this work can support other countries in their own price negotiations by setting realistic expectations for what hepatitis C elimination programs can achieve.

## Methods

### Model description

We used the Burnet hepatitis C model, which is described in detail elsewhere^2^^,^^16^ and has been used to perform regular projection updates of the Australian epidemic.^13^^,^^17^ In brief, the model classifies the Australian population according to risk group (people who inject drugs [PWID], former PWID and the general population), infection state (susceptible [Ab-/RNA- or Ab+/RNA-] or chronically infected), disease stage (F0 to F4, decompensated cirrhosis, hepatocellular carcinoma) and stage of the care cascade (undiagnosed, diagnosed Ab+ only, diagnosed RNA+, on treatment or failed treatment). For each time step people in the model can become infected according to a dynamic infection probability, move through the care cascade due to testing and treatment, develop more advanced liver disease, or die due to hepatitis C-related, injecting related or all-cause mortality. People in the model can commence, cease or relapse into injecting drug use, and the model is calibrated to population, epidemiological and clinical data from Australia (Table 3).

A key feature of the model is that people need to be diagnosed to access treatment. This means that the number of people initiating treatment per year in the model is constrained by both the number of diagnosed people available to initiate treatment (which is determined by the model inputs for number of tests and the test positivity rate) and the model input for total treatments available.

### Epidemiological inputs

Testing and treatment data inputs were taken from the Australian Medicare Benefits Schedule^18^ and the Pharmaceutical Benefits Scheme,^19^ respectively. All Australians are eligible for government subsidised testing and treatment, which is recorded in these systems. The main exception is for testing that occurs within the prison sector or from private providers. The cascade of care prior to introduction of DAA therapy was calibrated based on the observed cascade of care in Australia at that time in people who inject drugs (PWID) (from a community-based study^20^) and among non-PWID (from the national surveillance reports^21^). Estimated hepatitis C prevalence, the distribution of liver disease, and annual number of hepatitis C-related deaths were obtained from Australian national surveillance reports.^12^^,^^21^ Almost all new infections in Australia occur among PWID, and the estimated annual number of new hepatitis C infections among PWID were taken from a recent review.^22^

### Cost inputs

The economic costs associated with hepatitis C antibody testing, RNA testing, treatment, disease management and lost productivity were calculated from a societal perspective (i.e. regardless of who pays). All costs are reported in 2016 Australian dollars (A$) as this was the year that the DAA investment was made and the starting year of the economic analysis, and are discounted at 3.5% per annum (based on reserve bank near term GDP growth projections;^23^ 0% and 7% tested in sensitivity analyses). The methods for each cost component are summarized below with further details in Table 1 and Table 3.

**Table 1:** Testing and Treatment Numbers up to 2019

Variable	Value	Source
**Antibody Tests**	Calibrated to fit notification data	Notification data sourced from The Kirby Institute^12^
**RNA Tests**	2013: 17,288 2014: 17,425 2015: 17,443 2016: 25,404 2017: 24,360 2018: 18,703 2019: 17,497	MBS data.^13^ RNA tests were allocated across population groups (e.g. PWID versus non-PWID) on the assumption that PWID were twice as likely to be tested as non-PWID, based on targeted programs. This was tested in the sensitivity analysis.
**Treatments**	2013: 3,540* 2014: 3,749* 2015: 7,326* 2016: 32,650^ 2017: 21,560^ 2018: 16,490^ 2019: 11,580^	*Kirby Institute.^12^ We assumed reduced treatment success rate prior to 2016 (∼50%).^31^ ^Australia's progress towards hepatitis C elimination^11^ Treatments were allocated across population groups (e.g. PWID versus non-PWID) on the assumption that PWID were twice as likely to be treated as non-PWID, based on targeted programs. This was tested in the sensitivity analysis.

The costs of antibody testing, RNA testing and treatment included commodity, human resource and overhead cost components and were drawn from a costing analysis of a randomized controlled trial comparing primary and tertiary treatment pathways for hepatitis C^24^. These costs included opportunity costs associated with patient loss to follow up. In this model, the unit costs of testing were also applied to negative tests, with estimated testing positivity rates modelled to decline over time with reducing community prevalence. People with early liver disease (F0-2) were assumed to be treated through primary-based care, and people with advanced liver disease (F3+) through hospital-based care. Drug costs for individual treatments were not considered between 2016 and 2020; instead, the total cost of treatment (A$1.2 billion) was allocated across the 5-year period. From 2021-2025, treatment costs of $12,500 per course were used, reducing to $5,000 per course from 2026-2030 based on 100 times the cost of generics in low and middle-income countries (LMICs). DAA cost between 2021-2025 and 2026-2030 was also varied in a sensitivity analysis from $5,000 and $1,000 to $25,000 and $10,000.

The costs of disease management were estimated for each disease stage based on clinical guidelines and consultation with clinicians as to the type and frequency of appointments and tests undertaken by patients.^16^^,^^25^

The economic cost of lost productivity due to absenteeism (hepatitis C-related sick days), presenteeism (people being less productive as a result of their illness) and premature deaths were calculated using the human capital approach.^6^^,^^26^ Years of potential productive life lost among people with hepatitis C before and after cure were calculated by multiplying estimated rates of absenteeism and presenteeism^27^ by the employment rate, with different rates of absenteeism and presenteeism applied for people with/without cirrhosis and pre/post cure,^28^ and a reduced employment rate used for PWID.^29^ Years of potential productive life lost due to premature deaths were calculated by dynamically tracking a population of people who died from hepatitis C from their age at death until the assumed retirement age of 60 years. Years of potential productive life lost were converted to economic outcomes using population-weighted average per capita GDP.

### Scenarios projected

Three scenarios were considered as described in Table 2.

1) **Counterfactual:** pre-2016 testing/treatment numbers maintained up to 2030, to estimate the health and economic outcomes if universal DAA access had not occurred.
2) **Status-quo:** actual testing/treatment numbers based on MBS/PBS data for 2016-2019 with trends in testing and treatment projected to continue up to 2030.
3) **Elimination:** the same as the status-quo for 2016-2019, with testing and treatment sufficiently scaled up between 2020 and 2030 to achieve the 2030 WHO elimination targets of an 80% reduction in annual incidence and a 65% reduction in annual mortality compared to 2015 levels.

**Table 2:** Scenarios projected

Scenario	Description	Testing inputs	Treatment inputs
S1: No DAAs (counterfactual)	If no additional government-investment had occurred.	**Ab testing** Calibrated to fit notification data. **RNA testing** 2016-2030: 17,000 RNA tests per year.	2016:2030: Continued pre-2016 trends of 3,500 per year (but switching to DAAs from 2016)
S2: continued status-quo	Best estimated projections up to 2030.	**Ab testing** Calibrated to fit notification data. **RNA testing** 2020-2030: 17,000 RNA tests per year.	2020-2030: 10,000 per year (continued decreasing trend that stabilises)
S3: elimination	S2 with testing/treatment numbers increased to reach the WHO 2030 elimination targets.	**Ab testing** Calibrated to fit notification data. **RNA testing** **2020-2030: Calculated in scenario. Minimum required to reach elimination targets**	2019-2020: 10,000 **2021-2030: Calculated in scenario. Minimum required to reach elimination targets**

Treatment uptake was assumed to be twice as likely among PWID as non-PWID in all projections, based on ease of identification and ongoing frequent testing^30^ (tested in a sensitivity analysis).

### Outcomes

For each scenario, the model was projected for the period 2016-2030 and the main outcomes extracted were the projected people with hepatitis C, hepatitis C incidence and prevalence among PWID, total quality-adjusted life years (QALYs), and total costs (testing, treatment, disease management and productivity loss).

The cost-effectiveness realised from the original price negotiation was calculated as the cost per QALY gained at 2030 for the status-quo scenario relative to the counterfactual scenario from a health system perspective (i.e. excluding productivity gains).

The cost-effectiveness of continued efforts to achieve hepatitis C elimination was calculated as the cost per QALY gained at 2030 for the elimination scenario relative to the status-quo from a health system perspective (i.e. excluding productivity gains).

The net economic benefit over time of investing in hepatitis C treatment was calculated as the difference in cumulative costs between the status-quo scenario and the counterfactual scenario from a societal perspective (i.e. including testing, treatment, disease management and lost productivity costs).^6^ Similarly, the net economic benefit of the elimination scenario over time was calculated as the difference in cumulative costs between the elimination scenario and the counterfactual scenario from a societal perspective.

Costs and QALYs were discounted at 3.5% per annum (0% and 7% tested in sensitivity analyses).

### Uncertainty and sensitivity analyses

A multivariate probabilistic uncertainty analysis was conducted as follows to estimate uncertainty intervals for outcomes. Model projections were run 100 times with model parameters (from Table 3, hepatitis C parameters, direct costs, health utilities and productivity loss parameters) drawn at random from uniform distributions between their individual uncertainty bounds or +/-25% their point estimates. The inter-quartile range of outputs are reported.

**Table 3:** Parameter estimates and data inputs for the hepatitis C model

Variables	Range	Sources
*Hepatitis C parameters*		
Spontaneous clearance	26%	Micallef et al.^32^
Duration of acute stage	12 weeks	Mondelli et al.^33^
Treatment effectiveness	95%	Lawitz et al., Poorded et al., Gane et al. 3, 4, 5
Annual transition probabilities		
*F0->F1*	10.4-13.0%	Thein et al.^34^ In the model, rates are calibrated between bounds to fit the distribution of liver disease and mortality over time.
*F1->F2*	7.5-9.6%	
*F2->F3*	10.9-13.3%	
*F3->F4*	10.4-12.9%	
*F4->DC*	3.0-9.2%	National Centre in HIV Epidemiology and Clinical Research.^35^ In the model, rates are calibrated between bounds to fit the distribution of liver disease and mortality over time.
*F4->HCC*	0.9%-3.8%	
*DC->HCC*	4.1-9.9%	
*DC->death*	7.4-20.2%	
*HCC->death*	54.5-67.6%	
*F4->DC (post cure)*	74% reduced risk	Nahon et al.,^36^ hazard ratio = 0.26 (0.17-0.39) post cure.
*DC->HCC (post cure)*	71% reduced risk	Nahon et al.,^36^ hazard ratio = 0.29 (0.13-0.43) post cure.
*DC->death (post cure)*	73% reduced risk	Nahon et al.,^36^ hazard ratio = 0.27 (0.18-0.42) for overall mortality following cure for patents with cirrhosis.
*HCC->death (post cure)*	73% reduced risk	
*Direct costs parameters*		
Ab testing		
*Cost of test*	A$15.65	MBS item number 69405.^18^
*Staff cost*	A$37.60	General practitioner appointment, MBS item number 23.^18^
*Positivity rate*	4.1%	4% based on Australian Collaboration for Coordinated Enhanced Sentinel Surveillance (ACCESS) (ACCESS) data.^37^ Assumed to decrease to 1% by 2030
RNA testing		
*Cost of test*	A$92.20	MBS #69499.^18^
*Staff cost*	A$37.60	General practitioner appointment, MBS #23.^18^
*Positivity rate*	40% pre-2016, assumed to decrease linearly to 10% by 2030 in status-quo and elimination scenarios.	Australian Collaboration for Coordinated Enhanced Sentinel Surveillance (ACCESS) (ACCESS) data.^37^ Sensitivity analysis used to compare if the positivity rate for RNA tests remained at 40% up to 2030, or if it declined to 5% (instead of 10%).
Treatment		
*Drug cost*	2016-2020: A$13,190 per DAA course 2021-2025: A$12,500 2026-2030: A$5,000	For 2016-2020, cost per DAA course was estimated as the total A$1.2 billion divided by 90,980 treatments (70,980 from 2016-2018 and an estimated 20,000 from 2019 to 2020 based on current trends). For 2021-2025, assuming approximate current price is maintained. For 2026-2030, based on 100 times the cost of generics in low and middle-income countries.
*Staff and other pathology costs*	Time varying: A$1,846 per course in 2016 linearly decreasing to A$1,166 per course in 2021	In 2016 costs include A$462.10 for screening pathology + 38%*A$422.90 non-specialist care human resources + 62%*A$1615.90 specialist care human resources + A$221.24 pharmacy costs.^24^ Changes over time are based on the percentage of treatments delivered in non-specialist care increasing from 38% in 2016 to 63% in 2018,^13^ and continuing to increase linearly up to 95% in 2021 (maintained from 2021 onwards).
Disease management		
*F0-2*	A$447	Scott et al..^25^ Average costs per person per year, including appointment costs and recommended tests.
*F3*	A$691	
*F4*	A$935	
*DC*	A$15,202	
*HCC*	A$10,760	
Discounting	3.5% per annum	Applied to direct costs, productivity losses and quality-adjusted life years.
*Health utilities*		
Acute infection	0.751 (0.718-0.785)	Saeed et al. systematic review and meta analysis^38^
F0-F2	0.751 (0.718-0.785)	
F3	0.751 (0.718-0.785)	
F4	0.671 (0.630-0.713)	
DC	0.602 (0.551-0.653)	
HCC	0.662 (0.595-0.730)	
*Population and epidemiological parameters*		
15-64 year old population size	15,867,004 at start of 2016	Australian Bureau of Statistics.^39^
PWID population size	2010: 75,830 2011: 76,140 2012: 76,420 2013: 76,670 2014: 76,890 2015: 77,090 2016: 77,270	Kwon et al.^40^
Additional injecting-related mortality	0.0235 per year	Mathers et al.^41^
Hepatitis C antibody prevalence		
*PWID*	2015: 51%	Heard et al.^42^
*General population*	1.2% at start of 2016	Hepatitis C Mapping Project National Report.^43^
Total people with chronic hepatitis C (RNA+)	2015: 188,690* 2016: 160,280* 2017: 143,580 2018: 129,640	Kirby Institute^12^ *Personal communication
Hepatitis C-related mortality	2009: 460 2015: 740 2018: 410	
Incidence	4,126 new infections in 2015	Palmer et al.^22^
*Productivity loss parameters*		
Employment rate		
*General Population*	65%	Participation in workforce, averaged over 2015-2019, Australian Bureau of Statistics.^39^
*PWID*	14%	Reported employment status averaged over 2015-2019, Illicit Drug Reporting System (IDRS).^29^
Lost productivity attributable to hepatitis C		
*Absenteeism*	1.85%	Dibonaventura et al.^27^ US study (Australian study not available). People with hepatitis C had 4.88% absenteeism versus 3.03% for people without hepatitis C.
*Presenteeism*	3.19%	Dibonaventura et al.^27^ US study. People with hepatitis C had 16.69% presenteeism versus 13.50% for people without hepatitis C.
Additional productivity losses for people with cirrhosis		
*Absenteeism*	2.79 times	Younossi et al.^28^ European study (Australian study not available).
*Presenteeism*	1.54 times	
Relative reduction in absenteeism following hepatitis C cure		
*Cirrhotic*	44%	Younossi et al.^28^
*Non-cirrhotic*	0%	
Relative reduction in presenteeism following hepatitis C cure		
*Cirrhotic*	11%	Younossi et al.^28^
*Non-cirrhotic*	20%	
Per capita gross domestic product	A$53,663	Organisation for Economic Co-operation and Development (OECD) data for Australia.^44^
Percentage of hepatitis C-related deaths occurring at different age brackets		WHO cause-specific disease burden estimates, 2016.^45^
*15-29 years*	0.2%	
*30-49 years*	7.5%	
*50-59 years*	16.4%	
*60+ years*	75.8%	

One-way sensitivity analyses were undertaken to determine the effect on outcomes if: the price of DAAs between 2021-2035 and 2026-2030 was either $5,000/$1,000 or $25,000/$10,000, compared to $12,500/$5,000; RNA test positivity rates (i.e. percentage of RNA tests, conducted on antibody-positive individuals, that return positive) either remain at estimated pre-2016 levels (40%) or decrease to 5% by 2030, compared to the point estimate of a decrease to 10% by 2030; years of productive life lost were converted to economic outputs at +/-10% of per capita GDP (compared to per capita GDP of A$53,663); the annual growth rate of the PWID population in the model was increased or decreased by 25%; or treatment uptake among PWID was either equal to or four times treatment uptake in the general population, compared to being double.

### Role of the funder

This study was funded by the Burnet Institute. Funders had no role in study design, interpretation of results or decision to publish.

## Results

### No treatment scale-up (counterfactual scenario)

**Figure 1:**
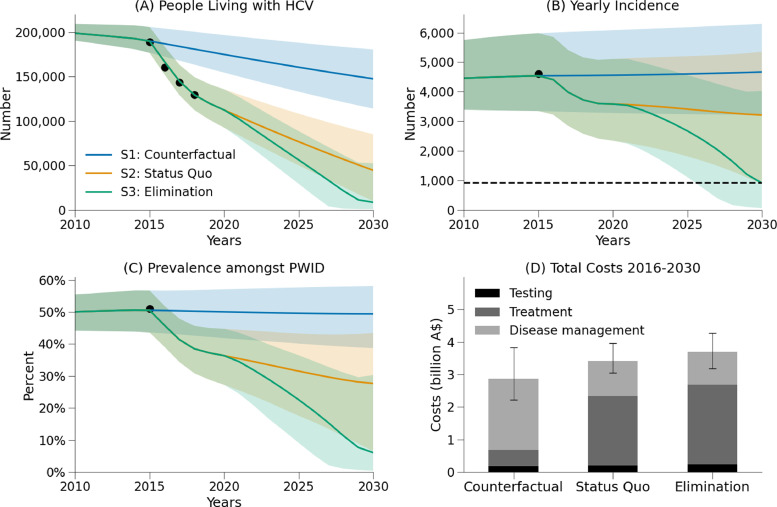
Outcomes for the counterfactual (S1, blue), status-quo (S2, orange) and elimination (S3, green) scenarios. (A) people with hepatitis C; (B) incidence; (C) prevalence among PWID; and (D) direct costs (testing, treatment, disease management).

Without treatment scale-up, the model projected that there would have been 147,400 people with hepatitis C in Australia in 2030, and an estimated 68,800 new hepatitis C infections and 18,540 hepatitis C-related deaths between 2016-2030 (Figure 1, blue).

This counterfactual scenario was estimated to cost $3.01 billion in direct health costs (testing, treatment and disease management) between 2016-2030, as well as A$26.14 billion in lost productivity due to absenteeism (hepatitis C-related sick days), presenteeism (people being less productive as a result of their illness) and premature deaths (Table 4).

**Table 4:** Model outcomes.

	S1: No DAAs	S2: Status-quo	S3: Elimination
**Costs (million Australian dollars)**			
Total direct costs	$3,007	$3,479	$3,722
	($2,431 - $3,890)	($3,167 - $3,857)	($3,282 - $4,136)
*Testing*	*$188*	*$213*	*$237*
	*($125 - $275)*	*($123 - $349)*	*($116 - $430)*
*Treatment*	*$519*	*$2,168*	*$2,479*
	*($519 - $519)*	*($2,142 - $2,168)*	*($2,349 - $2,504)*
*Disease management*	*$2,300*	*$1,098*	*$1,006*
	*($1,700 - $3,215)*	*($827 - $1,473)*	*($752 - $1,346)*
Lost productivity costs	$26,135	$19,963	$19,448
	($14,907 - $41,715)	($11,800 - $31,169)	($11,526 - $30,401)
*Absenteeism + presenteeism*	*$4,417*	*$4,173*	*$4,128*
	*($4,044 - $4,772)*	*($3,821 - $4,550)*	*($3,780 - $4,510)*
*Premature deaths*	*$21,718*	*$15,790*	*$15,320*
	*($10,371 - $37,510)*	*($7,514 - $27,125)*	*($7,284 - $26,362)*
**Difference in costs (million Australian dollars)**			
Total direct costs		$472	$715
		(-$100 - $858)	($44 - $1,194)
*Testing*		$25	$49
		($1 - $75)	($1 - $157)
*Treatment*		$1649	$1,960
		($1,623 - $1,649)	($1,830 - $1,985)
Disease management		-$1202	-$1,294
		(-$1,742 - -$853)	(-$1,881 - -$917)
Productivity gains		$6,172	$6,687
		($3,165 - $10,310)	($3,442 - $11,108)
*Absenteeism + presenteeism*		$244	$289
		($222 - $270)	($261 - $314)
*Premature deaths*		$5,928	$6,398
		($2,917 - $10,084)	($3,152 - $10,841)
**Cost-effectiveness**			
Total QALYs	221.76	221.84	221.86
	(221.66 - 221.86)	(221.73 - 221.96)	(221.74 - 221.97)
Cost per QALY gained at 2030 (compared with counterfactual scenario)		$5,752	$7,270
		(-$1,273 - $12,672)	($295 - $12,913)
Cost per QALY gained at 2030 (compared with status-quo			$12,150
			($4,869 - $26,532)
**Net economic benefit**			
At 2030 (millions)		$5,700	$5,972
		($2,376 - $10,190)	($2,356 - $10,836)
**Tests and treatment**			
Total number of antibody tests	3,194,000	3,566,000	3,739,000
	(1,912,000 - 4,944,000)	(1,708,437 - 6,329,000)	(1,604,000 - 7,909,000)
Total number of RNA tests	255,000	273,000	413,000
	(255,000 - 255,000)	(273,000 - 273,000)	(239,000 - 413,000)
Total number of treatments	47,700	181,300	210,800
	(47,700 - 47,700)	(175,300 - 181,300)	(182,800 - 216,100)
**Epidemiology**			
People with hepatitis C in 2030	147,400	44,500	8,500
	(113,900 - 180,400)	(9,900 - 85,000)	(700 - 52,500)
New infections 2015	4,537	4,536	4,536
	(3,344 - 5,980)	(3,343 - 5,979)	(3,343 - 5,979)
New infections 2030	4,665	3,212	906
	(3,224 - 6,294)	(874 - 5,352)	(59 - 4,023)
HCV-related deaths 2015	786	786	786
	(345 - 1,496)	(345 - 1,495)	(345 - 1,495)
HCV-related deaths 2030	1,424	362	219
	(806 - 2,063)	(187 - 548)	(106 - 383)
New infections 2016-2030	68,800	53,100	43,100
	(48,900 - 92,100)	(30,000 - 78,500)	(23,400 - 71,300)
HCV-related deaths 2016-2030	18,540	10,040	9,110
	(9,360 - 29,540)	(5,150 - 16,050)	(4,650 - 14,670)
HCV-prevalence among PWID in 2030 (%)	49%	28%	6%
	(39% - 58%)	(7% - 43%)	(0% - 30%)
HCV-prevalence among the whole population in 2030 (%)	0.89%	0.27%	0.05%
	(0.68% - 1.08%)	(0.06% - .51%)	(0.00% - 0.31%)
Cases averted compared to counterfactual		15,700	25,700
		(11,900 - 19,900)	(18,900 - 29,100)
Deaths averted compared to counterfactual		8,500	9,430
		(4,300 - 14,000)	(4,780 - 15,380)
**Progress towards targets**			
Reduction in incidence by 2030 (compared to 2015 levels)	-3%	29%	80%
	(-7% - 3%)	(9% - 74%)	(31% - 98%)
Reduction in mortality by 2030 (compared to 2015 levels)	-81%	54%	72%
	(-136% - -32%)	(29% - 78%)	(53% - 84%)

### The success of the current program (status-quo scenario)

Scaling up testing and treatment has had a major health impact in Australia. If current trends in testing and treatment were to continue, the model estimates that Australia will reduce the number of people with hepatitis C in 2030 from a projected 147,400 to 44,500, and avert a cumulative 8,500 hepatitis-C related deaths (46%) and 15,700 new infections (23%) over the period 2016-2030.

Scaling up testing and treatment has also been highly cost-effective with major economic benefits. The status-quo scenario is estimated to cost a total A$3.48 billion in direct health costs between 2016-2030, which is $472 million more than the counterfactual scenario. The status-quo scenario also includes a shift in where direct costs are incurred compared to the counterfactual, with A$1.65 billion more spent on treatment but A$1.20 billion less required for disease management (Figure 1). From a health system perspective (i.e. direct costs only), treatment scale-up is estimated to have had an incremental cost-effectiveness ratio of $5,752 per QALY gained at 2030.

**Figure 2:**
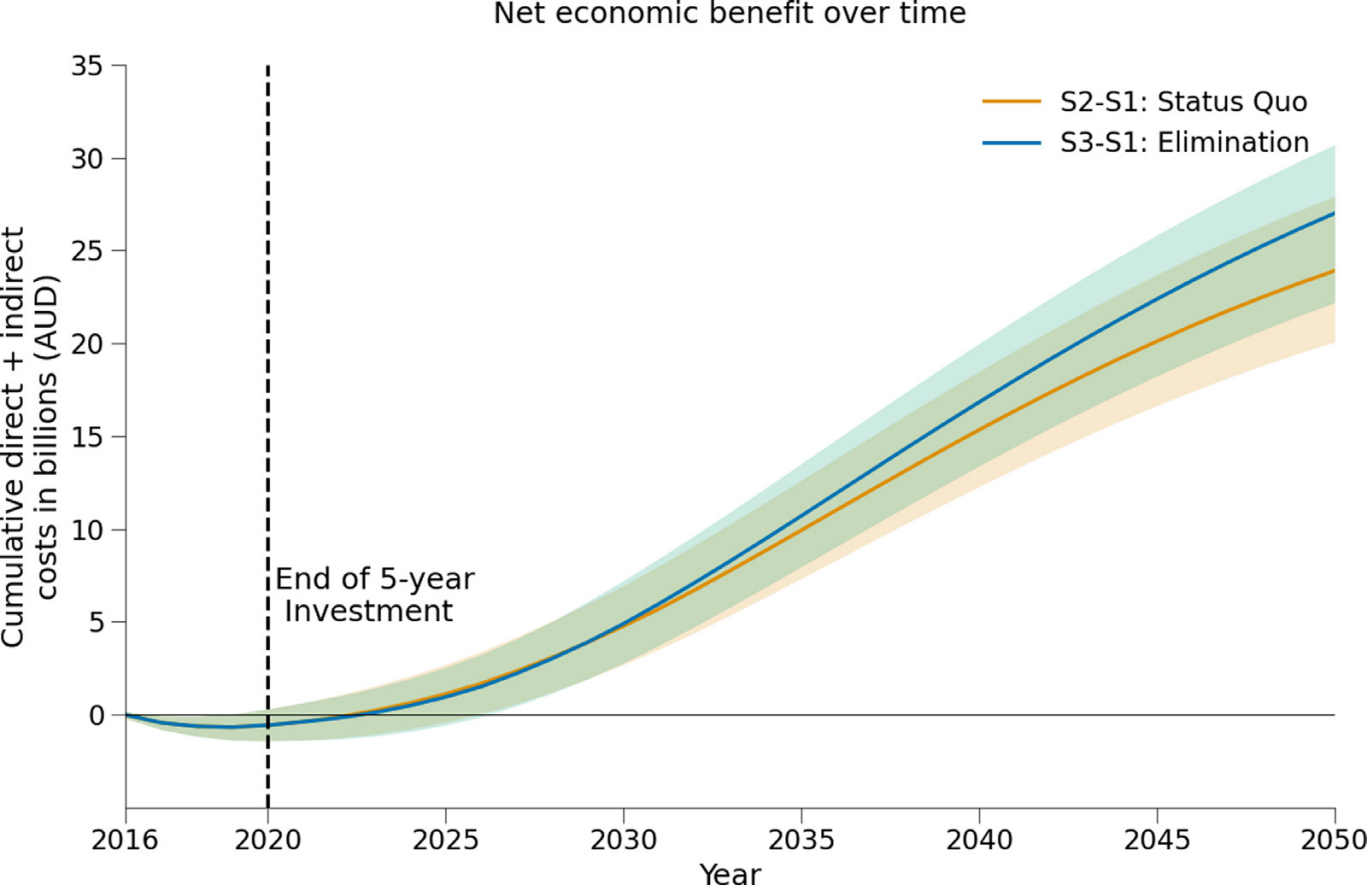
Net economic benefits of hepatitis C treatment scale-up. Orange: difference in cumulative costs (testing, treatment, disease management and productivity losses) between the status-quo and a scenario with no treatment scale-up. Green: difference in cumulative costs between the elimination scenario and a scenario with no treatment scale-up.

The status-quo is also on track to produce $6.17 billion in economic productivity gains between 2016-2030, compared to if treatment had remained at pre-2016 levels (counterfactual scenario). Due to these productivity gains, **the investment in hepatitis C treatment in Australia is estimated to become cost-saving in 2022, with a net economic benefit of $5.70 billion by 2030 (**Figure 2**).**

### Further efforts to achieve elimination (elimination scenario)

**Figure 3:**
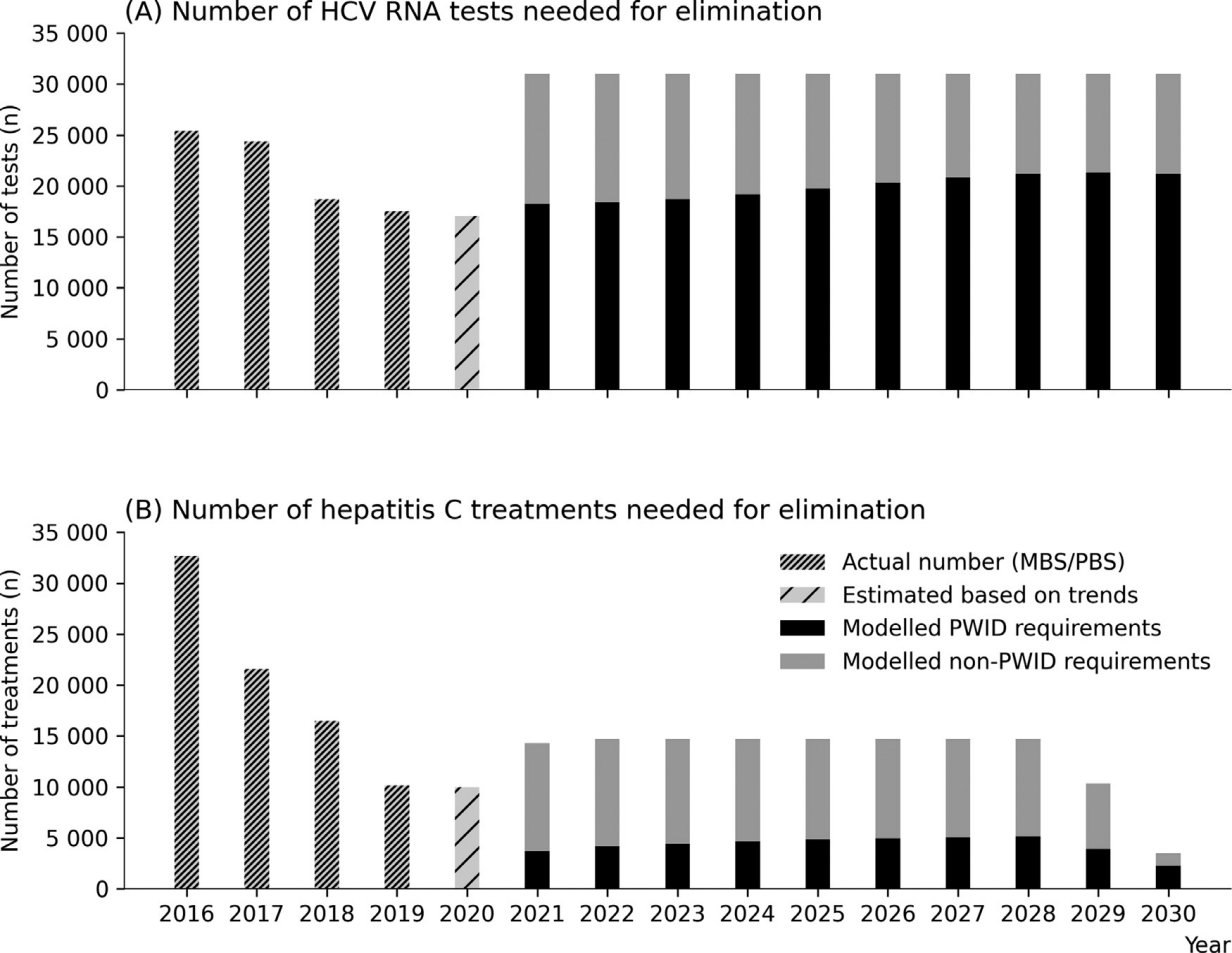
Model projections for the annual number of hepatitis C RNA tests (A) and hepatitis C treatments (B) needed between 2021-2030 to achieve the WHO elimination target of an 80% reduction in incidence by 2030, relative to 2015 levels. PWID refers to people who are currently injecting drugs.

In order to reach the hepatitis C elimination targets, treatment numbers would need to increase back to approximately 14,700 per annum (2018 levels). To achieve this, testing would need to be increased sufficiently to maintain high diagnosis rates: the model estimates that the annual number of RNA tests would need to increase from 17,000 per annum under the status-quo to approximately 31,000 per annum (Figure 3).

Reaching the incidence reduction target also requires that a sufficient number of treatments are among PWID in order to achieve treatment-as-prevention benefits. Assuming that between 2016-2020 HCV-infected PWID were twice as likely to be treated than HCV-infected non-PWID, this would imply treatment numbers among PWID decreasing from 6,300 in 2016 to 2,300 in 2020. To achieve elimination, it was estimated that treatments among PWID need to be increased to 4,200-5,400 per annum from 2021 onwards. Importantly, this number of treatments reflects a larger scale-up in earlier years (2021-2027); since annual new infections among PWID are estimated to be ∼3500 in 2020 (Figure 1B), treatment numbers among PWID must be sufficiently greater than this to ensure that prevalence among PWID continues to decline and treatment-as-prevention benefits are realised.

Further scale-up of testing and treatment to achieve the elimination targets could avert a *cumulative* additional 930 hepatitis C-related deaths and 10,000 new infections in Australia between 2021-2030 compared to continuing the status-quo (a total 9,430 deaths and 25,700 infections averted compared with no treatment scale-up).

The elimination scenario was estimated to cost a total $3.72 billion in direct health costs between 2016-2030, an additional $243 million more than the status-quo (an additional $335 million for testing/treatment and $92 million in disease costs averted). From a health systems perspective (i.e. direct costs only) this was estimated to have an incremental cost-effectiveness ratio of $12,150 per QALY gained compared to continuing the status-quo.

The elimination scenario was projected to increase the longer-term net economic benefit from $5.70 billion at 2030 under the status-quo to $5.92 billion with elimination (Figure 2 and Table 4).

## Discussion

The introduction of unrestricted DAA access in Australia in 2016 has led to substantial treatment uptake and major health and economic benefits. Compared to a scenario without treatment scale-up, we estimated that Australia is on track to avert 15,700 new infections and 8,500 hepatitis C-related deaths between 2016 and 2030. In addition to these massive improvements in individual health, the introduction of DAAs estimated to have become cost saving from a societal perspective by 2022, and to generate $5.70 billion in net economic benefits by 2030. However, consistent with previous analyses,^13^ we found that Australia will need to increase testing to achieve the WHO elimination targets. If this could be achieved and the elimination targets met, an additional 10,000 infections and 930 deaths could be averted.

This analysis provides new insights into the fundamental issue of the affordability of the public health response to hepatitis C, based on evidence from five years of a national elimination program. The model projections suggest that Australia's response to hepatitis C may have already become cost-saving from a societal perspective and has been highly cost-effective from a health systems perspective at $5,752 per QALY gained. The model also demonstrates how scaling up testing and treatment creates a major shift in the types of costs that are incurred. For example, compared to a scenario where treatments were maintained at pre-2016 levels, between 2016-2030 Australia is estimated to spend an additional A$1.65 billion on testing and treatment, but doing this is expected to save A$1.20 billion in disease management costs. As well as averting disease costs, curing hepatitis C also produces A$6.17 in economic benefits for society due to increased workforce participation, both among people who are cured and from the prevention of premature deaths.^27^^,^^28^^,^^46^^,^^47^ By adding these together, we estimate that the current response is on track to become cost saving from a societal perspective by 2021 and generate a net economic benefit of $5.70 billion by 2030.

These projections suggest that continuing the status-quo is likely to reduce the size of the epidemic but fall short of the WHO target for an 80% reduction in HCV incidence. Previous work has identified that following the introduction of DAAs, sub-optimal rates of diagnosis have been a major contributing factor to declining treatment numbers.^13^ By increasing testing in the model for the period 2021-2030 it was possible to increase treatments, with the model estimating that approximately 29,000 RNA tests and 14,700 treatments per annum would be required to achieve the WHO incidence target. However, reaching the incidence reduction target is more dependent on how treatments are targeted than total treatment numbers, and the model estimates that the necessary condition is treating 4200-5400 PWID per annum over 2021-30. In our main analysis, we assumed PWID with hepatitis C were twice as likely to be tested and treated as people with hepatitis C who do not inject drugs, based on ease of identification and ongoing frequent testing. This assumption, combined with population size and prevalence estimates, means that treating the required number of PWID corresponds to a total of approximately 14,700 treatments per annum over 2021-2030. It is difficult to tell what proportion of treatments have gone to PWID so far, because only data on total numbers are available. However, if a greater proportion of treatments were to go to PWID in the future, then the elimination target could be achieved faster or with fewer total treatments (Table S2). Reaching the elimination targets by increasing testing and treatment to these levels was estimated to modestly increase the total direct costs by an additional $243 million compared to continuing the status-quo, suggesting that it is likely to be affordable for Australia. Moreover, by further reducing ongoing transmission and perpetual treatment costs associated with these new infections, the net economic benefit was estimated to be $272 million greater in 2030, and the benefits compared to the status-quo would continue to increase every year thereafter.

The treatment targets estimated in this study are consistent with earlier analyses. Previously it was estimated that approximately 4,725 PWID per year would need to be treated each year over 2016-30 to achieve the WHO incidence target.^2^ If the same assumption that PWID are twice as likely to be treated as the general community is applied, this corresponds to approximately 14,000 treatments per annum over 2016-30 (based on approximately 189,000 people with hepatitis C and 38,500 HCV-infected PWID in 2015 [∼77,000 PWID and ∼50% prevalence]; meaning 12% annual uptake among PWID and 6% annual uptake among the rest of the population). More recently it was estimated that a scenario of 13,680 treatments per annum over 2019-30 would be sufficient to achieve the WHO incidence reduction target by 2026^51^. However, the assumptions about treatment uptake among PWID cannot be directly compared because a PWID sub-population or dynamic transmission were not included in that study. Another study has also estimated that approximately 30,000 RNA tests per annum would be required over 2020-30 to reach the incidence reduction target,^13^ roughly consistent with the scale-up in testing estimated here.

Our projections suggest that the WHO target for a 65% reduction in HCV-related mortality is unlikely to be met by continuing the status-quo, which is consistent with previous studies but should be interpreted with some degree of caution. Previous work suggests that meeting this target depends on assumptions about disease progression and liver-related mortality following cure. In^16^ it was found that the WHO target of a 65% reduction in hepatitis C-related mortality (among both infected and cured individuals) would only be met if disease progression and mortality was reduced by >54% post cure. In,^48^ using a base estimate of a 50% relative reduction in liver-related mortality following cure, the authors found that in a scenario of 13,680 treatments per annum a 65% reduction in mortality by 2030 could not be achieved overall (but could be achieved among just viremic individuals). In a sensitivity analysis the authors found that the overall mortality target could be met either in 2030 with an assumed 80% relative reduction in liver-related mortality following cure, or in 2023 with a more optimistic treatment scenario (∼21,000 per annum). By comparison, our study uses a 73% reduction in liver-related mortality following cure (^36^ and Table 3) and finds that the overall mortality target is not reached in the status quo but could be reached if treatments were to increase to 14,700 per annum. In our sensitivity analysis, we see that this is no longer the case if a 50% reduction in liver-related mortality following cure were assumed (Table S2). Together, these findings suggests that further work is required to understand the relative reduction in mortality risk following cure for people with advanced liver disease.

This study has relevance for other high-income countries. Australia was one of the first countries to introduce broad access to DAAs, and despite implementing a “best case scenario” of unrestricted DAA access, consisting of minimal patient costs, primary care prescribing, and prior investment in testing to begin with a significant number of people diagnosed, declines in treatment uptake have been observed. It is consistent with there being different phases in a country's hepatitis C elimination efforts.^49^ After the early adopters of treatment uptake are cured and hepatitis C becomes less prevalent, additional investment is required in outreach testing, including new approaches to enhance engagement with those who require extra support to access treatment, those who are hesitant about treatment and those who remain unaware that DAAs are available. Extra effort is required to support the affected communities, educate health care providers and simplify testing and treatment. These issues and lessons being learnt in Australia are likely to apply to other high-income countries with similar epidemics and health systems. The recent decline in treatment uptake has meant that fewer treatments were delivered than may have been hoped for in the initial five-year “capped cost” period if the first few years of treatment uptake had been sustained. This is where greater economic benefit is derived from, namely treating as many people as quickly as possible, which means that the net economic benefit reaches the break-even point earlier (Figure 2). A relatively quick break-even point has been possible in Australia because a large proportion of people with hepatitis C were already diagnosed, an essential component for other countries considering negotiating similar capped price arrangements for unlimited DAA courses.

The consideration of productivity loss related to hepatitis C is relevant beyond just high-income countries. Where the provision of hepatitis C care is limited due to limited resources, competing priorities or high rates of patient loss to follow-up, the introduction of testing and treatment will create new costs that will not necessarily be offset by healthcare cost savings as was estimated in Australia. Many economic analyses of hepatitis C testing and treatment do not consider the costs of lost productivity associated with hepatitis C, because these costs are borne by society rather than directly by the health system. However, even with conservative estimates (e.g. 1.85% additional absenteeism and 3.19% additional presenteeism;^27^ or approximately 4 additional sick days and 8 additional unproductive work days per person with hepatitis C per year), these costs to society are significant because of the large number of people for whom they apply. It follows that there are additional benefits to society from hepatitis C treatment in the form of productivity gains, and in this study we have estimated them following recent global analyses.^6^, ^7^, ^8^ These benefits are applicable even in settings where hepatitis C care is limited.

### Limitations

There are a number of limitations associated with model parameters. Model inputs, including epidemiological data, population data, health utilities and cost estimates come from a variety of sources and each have their own uncertainty. We conducted multivariate probabilistic uncertainty analyses on outcomes to attempt to capture this uncertainty in our confidence intervals. The parameters with the most significant impact on the net economic benefits at 2030 were identified as the cost of a year of productive life (based on per capita GDP), the assumed mortality reduction following cure, and the future cost of DAAs. Univariate sensitivity analyses have been used to show the impact that these parameters can have on outcomes when they vary between their upper and lower bound, and these sensitivity analyses are important when interpreting results.

There are also a number of limitations related to the specific scenarios. Each scenario relied on future estimates of testing and treatment, which are unknown. They also relied on estimated test positivity rates, which have implications for projected costs (although testing was a small component of overall costs). In particular, the estimate of $335 million extra in testing/treatment costs for the elimination scenario does not include the costs associated with demand generation activities to increase testing, because it is currently unclear what interventions would be required to increase testing rates. This is an important area for further work, as well as investigation of how testing can be incorporated in other models of care in a sustainable way.

Only one estimate for the annual number of new hepatitis C infections was available for model calibration, which was from 2015 based on a pooled analysis of incidence studies among PWID. Other estimates come from modelling studies, or incidence estimates that are based on biased subpopulations (e.g. NSP clients who are offered testing and treatment). This means that it is unclear how these projections for new hepatitis C infections are tracking against the real world. The model also only considered transmission among PWID, and not transmission among other groups such as HIV-positive men who have sex with men and mother-to-child transmission, however this is believed to be considerably lower in Australia due to high engagement in care and treatment uptake in these groups.^50^

## Conclusions

Unrestricted access to DAAs in Australia has led to significant health and economic benefits, with hepatitis C treatment scale-up on track to avert 15,700 new infections, 8,500 hepatitis C-related deaths and from a societal perspective become cost-saving by 2022 with a net economic benefit of $5.70 billion by 2030. Rapid treatment uptake at a known cost under a risk-sharing model was critical at achieving these early health and economic gains, yet will not be sufficient to meet our elimination targets. If Australia is to achieve the WHO elimination targets, testing and treatment needs to be increased, with a particular focus on treating 4,200-5,400 PWID per annum. Doing so is likely to avert an additional 10,000 infections and 930 hepatitis C-related deaths, and increase the net economic benefit at 2030 by $272 million.

## Authors’ contributions

NS, DW and MH conceived the study. NS designed the analyses and drafted the manuscript. APa set up and ran the scenarios. NS and TT developed the model. MS, RSD, JD, APe, AT, DW and MH sourced and validated model inputs. All authors were involved in revising the manuscript.

## Data sharing statement

Model parameters are available in tables and supplementary materials.

## Declaration of Competing Interest

The Burnet Institute receives funding from Gilead Sciences and Abbvie for investigator-initiated research unrelated to this work. JD is an advisory board member for Gilead Sciences, AbbVie and Merck and has received funding from Gilead Sciences and Abbie for investigator-initiated research unrelated to this work. APe has received investigator-initiated research funding from Gilead Sciences and AbbVie, and honoraria from Gilead Sciences. AT is an advisory board member and speaker for Gilead Sciences, AbbVie, Bristol-Myers Squibb (BMS) and Merck, and is on the Board of Directors (honorary) for the Gastroenterological Society of Australia. MS and MH have received funding from Gilead Sciences and Abbie for investigator-initiated research unrelated to this work. NS, APa, TT, RDS and DW have nothing to declare. No pharmaceutical grants were received in the development of this study.

## Appendix Supplementary materials

Image, application 1

## References

[1] World Health Organization. Global health sector strategy on viral hepatitis 2016-2021. Accessed 1 May 2018 from: http://www.who.int/hepatitis/strategy2016-2021/ghss-hep/en/. 2016.

[2] Scott N, McBryde E, Thompson A, Doyle J, Hellard M. (2017). Treatment scale-up to achieve global HCV incidence and mortality elimination targets: a cost-effectiveness model. Gut.

[3] Lawitz E, Poordad FF, Pang PS (2014). Sofosbuvir and ledipasvir fixed-dose combination with and without ribavirin in treatment-naive and previously treated patients with genotype 1 hepatitis C virus infection (LONESTAR): an open-label, randomised, phase 2 trial. The Lancet.

[4] Poordad F, Lawitz E, Kowdley KV (2013). Exploratory study of oral combination antiviral therapy for hepatitis C. New England Journal of Medicine.

[5] Gane EJ, Stedman CA, Hyland RH (2014). Efficacy of nucleotide polymerase inhibitor sofosbuvir plus the NS5A inhibitor ledipasvir or the NS5B non-nucleoside inhibitor GS-9669 against HCV genotype 1 infection. Gastroenterology.

[6] Scott N, Kuscel C, Pedrana A (2020). A model of the economic benefits of global hepatitis C elimination: an investment case. Lancet Gastroenterology and Hepatology.

[7] Pedrana A, Howell J, Scott N (2020). An investment framework for global hepatitis C elimination. Lancet Gastroenterology and Hepatology.

[8] Pedrana A, Howell J, Schröder S (2018). Eliminating Viral Hepatitis: The Investment Case. Doha, Qatar: World Innovation Summit for Health.

[9] Australian Pharmaceutical Benefits Scheme. General Statement for Drugs for the Treatment of Hepatitis C. Accessed 1 May 2018 from: http://www.pbs.gov.au/healthpro/explanatory-notes/general-statement-pdf/general-statement-hepatitis-c.pdf. 2016.

[10] Thompson A. (2016). Australian recommendations for the management of hepatitis C virus infection: a consensus statement. The Medical journal of Australia.

[11] Burnet Institute and Kirby Institute (2020).

[12] Kirby Institute. National update on HIV, viral hepatitis and sexually transmissible infections in Australia: 2009–2018. Sydney: Kirby Institute, UNSW Sydney. 2020.

[13] Scott N, Sacks-Davis R, Wade A (2019). Australia will need to increase testing to achieve hepatitis C elimination. Medical Journal of Australia.

[14] Scott N, Hainsworth SW, Sacks-Davis R (2018). Heterogeneity in hepatitis C treatment prescribing and uptake in Australia: a geospatial analysis of a year of unrestricted treatment access. Journal of Virus Eradication.

[15] Doyle J, Scott N, Sacks-Davis R (2019). Treatment access is only the first step to hepatitis C elimination: experience of universal anti-viral treatment access in Australia. Alimentary pharmacology & therapeutics.

[16] Scott N, Doyle J, Wilson DP (2017). Reaching hepatitis C virus elimination targets requires health system interventions to enhance the care cascade. International Journal of Drug Policy.

[17] Burnet Institute and Kirby Institute. Australia's progress towards hepatitis C elimination: annual report 2019. Melbourne: Burnet Institute; 2019.

[18] Australian Government Department of Human Services. Medicare Item Reports. Accessed 12 December 2018: http://medicarestatistics.humanservices.gov.au/statistics/mbs_item.jsp. 2018.

[19] Commonwealth of Australia Department of Health. Schedule of Pharmaceutical Benefits. Accessed 12 December 2018 from http://www.pbs.gov.au/.

[20] Wade AJ, Macdonald DM, Doyle JS (2015). The Cascade of Care for an Australian Community-Based Hepatitis C Treatment Service. PLoS One.

[21] The Kirby Institute. HIV, viral hepatitis and sexually transmissible infections in Australia: annual surveillance report 2018. Sydney: Kirby Institute, UNSW Sydney. Accessed 16 August 2019 from: https://kirby.unsw.edu.au/report/hiv-viral-hepatitis-and-sexually-transmissible-infections-australia-annual-surveillance. 2018.

[22] Palmer AY, Wilkinson A, Aitken C (2021). Estimating the number of new hepatitis C infections in Australia in 2015, prior to the scale-up of direct-acting antiviral treatment. Journal of Gastroenterology and Hepatology.

[23] Reserve Bank of Australia. February 2021 forecast table. Accessed 29 March 2021 from: https://www.rba.gov.au/publications/smp/2021/feb/forecasts.html. 2021.

[24] Palmer AY, Wade AJ, Draper B (2020). A cost-effectiveness analysis of primary versus hospital-based specialist care for direct acting antiviral hepatitis C treatment. International Journal of Drug Policy.

[25] Scott N, Iser D, Thompson A, Doyle J, Hellard M. (2016). Cost-effectiveness of treating chronic hepatitis C virus with direct-acting antivirals in people who inject drugs in Australia. Journal of Gastroenterology and Hepatology.

[26] Grossman M. (1972). On the concept of health capital and the demand for health. Journal of Political Economy.

[27] Dibonaventura MD, Wagner J-S, Yuan Y, L'Italien G, Langley P, Ray Kim W. (2011). The impact of hepatitis C on labor force participation, absenteeism, presenteeism and non-work activities. Journal of Medical Economics.

[28] Younossi Z, Brown A, Buti M (2016). Impact of eradicating hepatitis C virus on the work productivity of chronic hepatitis C (CH-C) patients: an economic model from five European countries. Journal of Viral Hepatitis.

[29] Peacock A, Gibbs D, Sutherland R, et al. Australian Drug Trends 2018. Key findings from the National Illicit Drug Reporting System (IDRS) Interviews. Accessed 16 August 2019 from: https://ndarc.med.unsw.edu.au/resource/australian-drug-trends-2018-key-findings-national-illicit-drug-reporting-system-idrs.*National Drug and Alcohol Research Centre, UNSW Australia* 2018.

[30] Memedovic S, Iversen J, Geddes L, Maher L. Australian Needle Syringe Program Survey National Data Report 2012-2016: Prevalence of HIV, HCV and injecting and sexual behaviour among NSP attendees. Sydney: Kirby Institute, UNSW Australia. Accessed 16 August 2019 from: https://kirby.unsw.edu.au/report/australian-nsp-survey-national-data-report-2012-2016. *ISSN: 1448-5915* 2017.

[31] Roberts SK, Weltman MD, Crawford DH (2009). Impact of high-dose peginterferon alfa-2A on virological response rates in patients with hepatitis C genotype 1: a randomized controlled trial. Hepatology.

[32] Micallef JM, Kaldor JM, Dore GJ. (2006). Spontaneous viral clearance following acute hepatitis C infection: a systematic review of longitudinal studies. Journal of Viral Hepatitis.

[33] Mondelli MU, Cerino A, Cividini A. (2005). Acute hepatitis C: diagnosis and management. Journal of hepatology.

[34] Thein HH, Yi Q, Dore GJ, Krahn MD. (2008). Estimation of stage-specific fibrosis progression rates in chronic hepatitis C virus infection: a meta-analysis and meta-regression. Hepatology.

[35] National Centre in HIV Epidemiology and Clinical Research. Epidemiological and economic impact of potential increased hepatitis C treatment uptake in Australia. *National Centre in HIV Epidemiology and Clinical Research, The University of New South Wales, Sydney, NSW*2010.

[36] Nahon P, Bourcier V, Layese R (2017). Eradication of hepatitis C virus infection in patients with cirrhosis reduces risk of liver and non-liver complications. Gastroenterology.

[37] Callander D, Moreira C, El-Hayek C (2018). Monitoring the control of sexually transmissible infections and blood-borne viruses: protocol for the Australian Collaboration for Coordinated Enhanced Sentinel Surveillance (ACCESS). JMIR research protocols.

[38] Saeed YA, Phoon A, Bielecki JM (2020). A systematic review and meta-analysis of health utilities in patients with chronic hepatitis C. Value in Health.

[39] Australian Bureau of Statistics (ABS). http://www.abs.gov.au/. 2021.

[40] Kwon JA, Iversen J, Law M, Dolan K, Wand H, Maher L. (2019). Estimating the number of people who inject drugs and syringe coverage in Australia, 2005–2016. Drug and alcohol dependence.

[41] Mathers BM, Degenhardt L, Bucello C, Lemon J, Wiessing L, Hickman M. (2013). Mortality among people who inject drugs: a systematic review and meta-analysis. Bulletin of the World Health Organization.

[42] Heard S, Iversen J, Geddes L, Maher L. Australian NSP survey: Prevalence of HIV, HCV and injecting and sexual behaviour among NSP attendees, 25-year National Data Report 1995-2019. Sydney: The Kirby Institute, UNSW Sydney. 2020.

[43] MacLachlan J, Thomas L, Cowie B, Allard N. Hepatitis C Mapping Project: Estimates of geographic diversity in chronic hepatitis C prevalence, diagnosis, monitoring and treatment - National Report 2016. 2018; **ISBN: 978-1-921850-28-8**.

[44] Organisation for Economic Co-operation and Development (OECD). https://data.oecd.org/.

[45] World Health Organization. Disease burden and mortality estimates; cause-specific mortality, 2000–2016. Available from: http://www.who.int/healthinfo/global_burden_disease/estimates/en/. 2016.

[46] Su J, Brook RA, Kleinman NL, Corey-Lisle P. (2010). The impact of hepatitis C virus infection on work absence, productivity, and healthcare benefit costs. Hepatology.

[47] Younossi Z, Chan H, Dan Y (2018). Impact of ledipasvir/sofosbuvir on the work productivity of genotype 1 chronic hepatitis C patients in Asia. Journal of Viral Hepatitis.

[48] Kwon JA, Dore GJ, Grebely J (2018). Australia on track to achieve WHO HCV elimination targets following rapid initial DAA treatment uptake: a modeling study. Journal of viral hepatitis.

[49] Pedrana A, Munari S, Stoové M, Doyle J, Hellard M. (2021). The phases of hepatitis C elimination: achieving WHO elimination targets. The Lancet Gastroenterology & Hepatology.

[50] Doyle J, van Santen D, Iser D (2020). Micro-elimination of hepatitis C among people with HIV coinfection: declining incidence and prevalence accompanying a multi-center treatment scale-up trial. Clinical Infectious Diseases: an Official Publication of the Infectious Diseases Society of America.

