# Supplementary material for: Assessment of the cost-effectiveness of Australia's risk-sharing agreement for direct-acting antiviral treatments for hepatitis C: a modelling study
Source: Lancet Reg Health West Pac. 2021 Nov 23;18:100316. doi: 10.1016/j.lanwpc.2021.100316 (PMC8669355; doi:10.1016/j.lanwpc.2021.100316)
Supplement: Supplementary file 1 [file mmc1.docx]

# Appendix A: sensitivity analysis

Table S1: Sensitivity analysis for counterfactual scenario (scenario 1, no treatment scale-up)

| **Scenario 1** | **PLHCV in 2030** | **New infections 2016-2030** | **Deaths 2016-2030** | **Incidence in 2030 relative to 2015 (%)** | **Mortality in 2030 relative to 2015 (%)** | **Total direct costs (million A$)** | **Lost productivity costs (million A$)** |
| --- | --- | --- | --- | --- | --- | --- | --- |
| ***Cost of DAAs 2021 onwards*** |  |  |  |  |  |  |  |
| Point estimate ($5,000 per treatment course) | 147,416 | 68,809 | 18,538 | 103 | 181 | 3,007 | 26,135 |
| $1,000 per treatment course | 147,416 | 68,809 | 18,538 | 103 | 181 | 2,851 | 26,135 |
| $10,000 per treatment course | 147,416 | 68,809 | 18,538 | 103 | 181 | 3,247 | 26,135 |
| ***RNA test positivity*** |  |  |  |  |  |  |  |
| Point estimate (decr. to 10% by 2030) | 147,416 | 68,809 | 18,538 | 103 | 181 | 3,007 | 26,135 |
| RNA test positivity remains constant | 147,384 | 68,580 | 18,614 | 102 | 184 | 3,081 | 26,166 |
| RNA test positivity decr. to 5% by 2030 | 147,422 | 68,849 | 18,524 | 103 | 181 | 2,993 | 26,129 |
| ***Targeting of PWID*** |  |  |  |  |  |  |  |
| Point estimate (PWID twice as likely to be tested/treated) | 147,416 | 68,809 | 18,538 | 103 | 181 | 3,007 | 26,135 |
| PWID equally as likely to be tested/treated | 147,416 | 68,809 | 18,538 | 103 | 181 | 3,007 | 26,135 |
| PWID 4 times as likely to be tested/treated | 147,416 | 68,809 | 18,538 | 103 | 181 | 3,007 | 26,135 |
| ***Reduction in mortality post cure*** |  |  |  |  |  |  |  |
| Point estimate (73% reduction) | 147,416 | 68,809 | 18,538 | 103 | 181 | 3,007 | 26,135 |
| 50% reduction | 147,415 | 68,810 | 18,846 | 103 | 184 | 3,007 | 26,374 |
| No reduction | 147,414 | 68,811 | 19,235 | 103 | 186 | 3,006 | 26,696 |
| ***GDP*** |  |  |  |  |  |  |  |
| Point estimate (A$53,663) | 147,416 | 68,809 | 18,538 | 103 | 181 | 3,007 | 26,135 |
| GDP decreased by 10% | 147,416 | 68,809 | 18,538 | 103 | 181 | 3,007 | 23,521 |
| GDP increased by 10% | 147,416 | 68,809 | 18,538 | 103 | 181 | 3,007 | 28,748 |
| ***Employment*** |  |  |  |  |  |  |  |
| Point estimate (General – 65%, PWID – 14%) | 147,416 | 68,809 | 18,538 | 103 | 181 | 3,007 | 26,135 |
| Employment decreased by 10% | 147,416 | 68,809 | 18,538 | 103 | 181 | 3,007 | 23,521 |
| Employment increased by 10% | 147,416 | 68,809 | 18,538 | 103 | 181 | 3,007 | 28,748 |
| ***Length of injecting career*** |  |  |  |  |  |  |  |
| Point estimate | 147,416 | 68,809 | 18,538 | 103 | 181 | 3,007 | 26,135 |
| Length of injecting career decreased by 25% | 143,818 | 64,777 | 18,540 | 88 | 181 | 3,007 | 26,181 |
| Length of injecting career increased by 25% | 149,785 | 71,476 | 18,536 | 113 | 181 | 3,006 | 26,102 |
| ***Force of infection*** |  |  |  |  |  |  |  |
| Point estimate | 147,416 | 68,809 | 18,538 | 103 | 181 | 3,007 | 26,135 |
| Force of infection decreased by 25% | 136,233 | 57,155 | 18,528 | 74 | 181 | 3,006 | 26,096 |
| Force of infection increased by 25% | 157,360 | 79,248 | 18,546 | 125 | 181 | 3,007 | 26,170 |
| ***PWID recruitment rate*** |  |  |  |  |  |  |  |
| Point estimate | 147,416 | 68,809 | 18,538 | 103 | 181 | 3,007 | 26,135 |
| PWID recruitment rate increased by 25% | 152,470 | 74,304 | 18,537 | 124 | 181 | 3,007 | 26,148 |
| PWID recruitment rate decreased by 25% | 144,034 | 65,129 | 18,539 | 89 | 181 | 3,007 | 26,126 |
| ***Discounting*** |  |  |  |  |  |  |  |
| Point estimate (3.5% discounting) | 147,416 | 68,809 | 18,538 | 103 | 181 | 3,007 | 26,135 |
| Discounting 0% | 147,416 | 68,809 | 18,538 | 103 | 181 | 3,298 | 29,741 |
| Discounting 7% | 147,416 | 68,809 | 18,538 | 103 | 181 | 2,801 | 23,531 |
| ***Testing costs*** |  |  |  |  |  |  |  |
| Point estimate | 147,416 | 68,809 | 18,538 | 103 | 181 | 3,007 | 26,135 |
| Testing costs halved (Ab and RNA) | 147,416 | 68,809 | 18,538 | 103 | 181 | 2,913 | 26,135 |
| Testing costs doubled (Ab and RNA) | 147,416 | 68,809 | 18,538 | 103 | 181 | 3,195 | 26,135 |
| ***Disease costs*** |  |  |  |  |  |  |  |
| Point estimate | 147,416 | 68,809 | 18,538 | 103 | 181 | 3,007 | 26,135 |
| Disease management costs halved | 147,416 | 68,809 | 18,538 | 103 | 181 | 1,857 | 26,135 |
| Disease management costs doubled | 147,416 | 68,809 | 18,538 | 103 | 181 | 4,157 | 26,135 |
| ***Health utilities when infected with hepatitis C*** |  |  |  |  |  |  |  |
| Point estimate | 147,416 | 68,809 | 18,538 | 103 | 181 | 3,007 | 26,135 |
| Health utilities are at their lower bound estimates | 147,416 | 68,809 | 18,538 | 103 | 181 | 3,007 | 26,135 |
| Health utilities are at their upper bound estimates | 147,416 | 68,809 | 18,538 | 103 | 181 | 3,007 | 26,135 |

Table S2: Sensitivity analysis for status-quo scenario (scenario 2, DAA scale-up as has occurred)

| **Scenario 2** | **PLHCV in 2030** | **New infections 2016-2030** | **Deaths 2016-2030** | **Incidence in 2030 relative to 2015 (%)** | **Mortality in 2030 relative to 2015 (%)** | **Total direct costs (million A$)** | **Lost productivity costs (million A$)** | **Cost per QALY gained (compared to S1)** | **Net economic benefit at 2030 (compared to S1)** | **Net economic benefit at 2050 (compared to S1)** |
| --- | --- | --- | --- | --- | --- | --- | --- | --- | --- | --- |
| ***Cost of DAAs 2021 onwards*** |  |  |  |  |  |  |  |  |  |  |
| Point estimate ($5,000 per treatment course) | 44,493 | 53,144 | 10,037 | 71 | 46 | 3,479 | 19,963 | 5,752 | 5,698 | 23,896 |
| $1,000 per treatment course | 44,493 | 53,144 | 10,037 | 71 | 46 | 3,013 | 19,963 | 1,979 | 6,009 | 24,151 |
| $10,000 per treatment course | 44,493 | 53,144 | 10,037 | 71 | 46 | 4,196 | 19,963 | 11,553 | 5,222 | 23,488 |
| ***RNA test positivity*** |  |  |  |  |  |  |  |  |  |  |
| Point estimate (decr. to 10% by 2030) | 44,493 | 53,144 | 10,037 | 71 | 46 | 3,479 | 19,963 | 5,752 | 5,698 | 23,896 |
| RNA test positivity remains constant | 41,787 | 50,352 | 10,406 | 54 | 58 | 3,586 | 20,120 | 6,115 | 5,542 | 25,085 |
| RNA test positivity decr. to 5% by 2030 | 47,189 | 53,765 | 9,957 | 75 | 43 | 3,442 | 19,931 | 5,477 | 5,748 | 23,324 |
| ***Targeting of PWID*** |  |  |  |  |  |  |  |  |  |  |
| Point estimate (PWID twice as likely to be tested/treated) | 44,493 | 53,144 | 10,037 | 71 | 46 | 3,479 | 19,963 | 5,752 | 5,698 | 23,896 |
| PWID equally as likely to be tested/treated | 48,339 | 59,386 | 9,414 | 80 | 40 | 3,428 | 19,505 | 5,531 | 6,208 | 24,901 |
| PWID 4 times as likely to be tested/treated | 36,844 | 44,549 | 10,702 | 55 | 51 | 3,534 | 20,469 | 5,767 | 5,139 | 22,998 |
| ***Reduction in mortality post cure*** |  |  |  |  |  |  |  |  |  |  |
| Point estimate (73% reduction) | 44,493 | 53,144 | 10,037 | 71 | 46 | 3,479 | 19,963 | 1,899 | 5,698 | 23,896 |
| 50% reduction | 44,491 | 53,146 | 10,952 | 71 | 56 | 3,479 | 20,686 | 1,908 | 5,215 | 23,303 |
| No reduction | 44,488 | 53,154 | 12,096 | 71 | 65 | 3,478 | 21,668 | 1,920 | 4,556 | 22,779 |
| ***GDP*** |  |  |  |  |  |  |  |  |  |  |
| Point estimate (A$53,663) | 44,493 | 53,144 | 10,037 | 71 | 46 | 3,479 | 19,963 | 5,752 | 5,698 | 23,896 |
| GDP decreased by 10% | 44,493 | 53,144 | 10,037 | 71 | 46 | 3,479 | 17,967 | 5,752 | 5,081 | 21,576 |
| GDP increased by 10% | 44,493 | 53,144 | 10,037 | 71 | 46 | 3,479 | 21,960 | 5,752 | 6,316 | 26,216 |
| ***Employment*** |  |  |  |  |  |  |  |  |  |  |
| Point estimate (General – 65%, PWID – 14%) | 44,493 | 53,144 | 10,037 | 71 | 46 | 3,479 | 19,963 | 5,752 | 5,698 | 23,896 |
| Employment decreased by 10% | 44,493 | 53,144 | 10,037 | 71 | 46 | 3,479 | 17,967 | 5,752 | 5,081 | 21,576 |
| Employment increased by 10% | 44,493 | 53,144 | 10,037 | 71 | 46 | 3,479 | 21,960 | 5,752 | 6,316 | 26,216 |
| ***Length of injecting career*** |  |  |  |  |  |  |  |  |  |  |
| Point estimate | 44,493 | 53,144 | 10,037 | 71 | 46 | 3,479 | 19,963 | 5,752 | 5,698 | 23,896 |
| Length of injecting career decreased by 25% | 40,197 | 49,795 | 10,024 | 58 | 46 | 3,478 | 19,997 | 5,755 | 5,713 | 24,103 |
| Length of injecting career increased by 25% | 47,417 | 55,400 | 10,046 | 80 | 46 | 3,480 | 19,939 | 5,752 | 5,688 | 23,750 |
| ***Force of infection*** |  |  |  |  |  |  |  |  |  |  |
| Point estimate | 44,493 | 53,144 | 10,037 | 71 | 46 | 3,479 | 19,963 | 5,752 | 5,698 | 23,896 |
| Force of infection decreased by 25% | 30,564 | 41,687 | 9,994 | 37 | 45 | 3,476 | 19,912 | 5,741 | 5,714 | 24,342 |
| Force of infection increased by 25% | 58,887 | 65,192 | 10,077 | 105 | 47 | 3,482 | 20,015 | 5,860 | 5,680 | 23,494 |
| ***PWID recruitment rate*** |  |  |  |  |  |  |  |  |  |  |
| Point estimate | 44,493 | 53,144 | 10,037 | 71 | 46 | 3,479 | 19,963 | 5,752 | 5,698 | 23,896 |
| PWID recruitment rate increased by 25% | 46,708 | 56,176 | 10,033 | 82 | 46 | 3,479 | 19,969 | 5,613 | 5,706 | 24,024 |
| PWID recruitment rate decreased by 25% | 42,972 | 51,054 | 10,040 | 63 | 46 | 3,480 | 19,960 | 5,846 | 5,694 | 23,816 |
| ***Discounting*** |  |  |  |  |  |  |  |  |  |  |
| Point estimate (3.5% discounting) | 44,493 | 53,144 | 10,037 | 71 | 46 | 3,479 | 19,963 | 5,752 | 5,698 | 23,896 |
| Discounting 0% | 44,493 | 53,144 | 10,037 | 71 | 46 | 3,657 | 22,147 | 3,773 | 7,235 | 43,187 |
| Discounting 7% | 44,493 | 53,144 | 10,037 | 71 | 46 | 3,353 | 18,419 | 7,615 | 4,559 | 14,281 |
| ***Testing costs*** |  |  |  |  |  |  |  |  |  |  |
| Point estimate | 44,493 | 53,144 | 10,037 | 71 | 46 | 3,479 | 19,963 | 5,752 | 5,698 | 23,896 |
| Testing costs halved (Ab and RNA) | 44,493 | 53,144 | 10,037 | 71 | 46 | 3,373 | 19,963 | 5,596 | 5,711 | 23,909 |
| Testing costs doubled (Ab and RNA) | 44,493 | 53,144 | 10,037 | 71 | 46 | 3,693 | 19,963 | 6,064 | 5,673 | 23,871 |
| ***Disease costs*** |  |  |  |  |  |  |  |  |  |  |
| Point estimate | 44,493 | 53,144 | 10,037 | 71 | 46 | 3,479 | 19,963 | 5,752 | 5,698 | 23,896 |
| Disease management costs halved | 44,493 | 53,144 | 10,037 | 71 | 46 | 2,930 | 19,963 | 13,068 | 5,097 | 22,755 |
| Disease management costs doubled | 44,493 | 53,144 | 10,037 | 71 | 46 | 4,028 | 19,963 | -1,563 | 6,300 | 25,037 |
| ***Health utilities when infected with hepatitis C*** |  |  |  |  |  |  |  |  |  |  |
| Point estimate | 44,493 | 53,144 | 10,037 | 71 | 46 | 3,479 | 19,963 | 5,752 | 5,698 | 23,896 |
| Health utilities are at their lower bound estimates | 44,493 | 53,144 | 10,037 | 71 | 46 | 3,479 | 19,963 | 4,108 | 5,698 | 23,896 |
| Health utilities are at their upper bound estimates | 44,493 | 53,144 | 10,037 | 71 | 46 | 3,479 | 19,963 | 9,767 | 5,698 | 23,896 |

Table S3: Sensitivity analysis for elimination scenario (scenario 3, testing and treatment scale-up to achieve elimination targets)

| **Scenario 3** | **PLHCV in 2030** | **New infections 2016-2030** | **Deaths 2016-2030** | **Incidence in 2030 relative to 2015 (%)** | **Mortality in 2030 relative to 2015 (%)** | **Total direct costs (million A$)** | **Lost productivity costs (million A$)** | **Cost per QALY gained (compared to S1)** | **Net economic benefit at 2030 (compared to S1)** | **Net economic benefit at 2050 (compared to S1)** |
| --- | --- | --- | --- | --- | --- | --- | --- | --- | --- | --- |
| ***Cost of DAAs 2021 onwards*** |  |  |  |  |  |  |  |  |  |  |
| Point estimate ($5,000 per treatment course) | 8,532 | 43,137 | 9,108 | 20 | 28 | 3,722 | 19,448 | 7,270 | 5,971 | 27,794 |
| $1,000 per treatment course | 8,532 | 43,137 | 9,108 | 20 | 28 | 3,081 | 19,448 | 2,346 | 6,455 | 28,182 |
| $10,000 per treatment course | 8,532 | 43,137 | 9,108 | 20 | 28 | 4,718 | 19,448 | 14,961 | 5,215 | 27,159 |
| ***RNA test positivity*** |  |  |  |  |  |  |  |  |  |  |
| Point estimate (decr. to 10% by 2030) | 8,532 | 43,137 | 9,108 | 20 | 28 | 3,722 | 19,448 | 7,270 | 5,971 | 27,794 |
| RNA test positivity remains constant | 2,022 | 40,083 | 9,348 | 6 | 28 | 3,762 | 19,591 | 6,791 | 5,894 | 28,506 |
| RNA test positivity decr. to 5% by 2030 | 19,972 | 45,149 | 9,046 | 39 | 30 | 3,666 | 19,411 | 6,945 | 6,044 | 26,431 |
| ***Targeting of PWID*** |  |  |  |  |  |  |  |  |  |  |
| Point estimate (PWID twice as likely to be tested/treated) | 8,532 | 43,137 | 9,108 | 20 | 28 | 3,722 | 19,448 | 7,270 | 5,971 | 27,794 |
| PWID equally as likely to be tested/treated | 15,578 | 51,494 | 8,541 | 37 | 26 | 3,718 | 18,998 | 7,902 | 6,425 | 28,307 |
| PWID 4 times as likely to be tested/treated | 1,163 | 32,379 | 9,670 | 3 | 28 | 3,696 | 19,926 | 6,255 | 5,520 | 27,207 |
| ***Reduction in mortality post cure*** |  |  |  |  |  |  |  |  |  |  |
| Point estimate (73% reduction) | 8,532 | 43,137 | 9,108 | 20 | 28 | 3,722 | 19,448 | 2,464 | 5,971 | 27,794 |
| 50% reduction | 8,527 | 43,139 | 10,127 | 20 | 37 | 3,721 | 20,233 | 2,475 | 5,426 | 27,192 |
| No reduction | 8,520 | 43,143 | 11,402 | 20 | 45 | 3,721 | 21,307 | 2,492 | 4,675 | 26,692 |
| ***GDP*** |  |  |  |  |  |  |  |  |  |  |
| Point estimate (A$53,663) | 8,532 | 43,137 | 9,108 | 20 | 28 | 3,722 | 19,448 | 7,270 | 5,971 | 27,794 |
| GDP decreased by 10% | 8,532 | 43,137 | 9,108 | 20 | 28 | 3,722 | 17,504 | 7,270 | 5,303 | 25,089 |
| GDP increased by 10% | 8,532 | 43,137 | 9,108 | 20 | 28 | 3,722 | 21,393 | 7,270 | 6,640 | 30,499 |
| ***Employment*** |  |  |  |  |  |  |  |  |  |  |
| Point estimate (General – 65%, PWID – 14%) | 8,532 | 43,137 | 9,108 | 20 | 28 | 3,722 | 19,448 | 7,270 | 5,971 | 27,794 |
| Employment decreased by 10% | 8,532 | 43,137 | 9,108 | 20 | 28 | 3,722 | 17,504 | 7,270 | 5,303 | 25,089 |
| Employment increased by 10% | 8,532 | 43,137 | 9,108 | 20 | 28 | 3,722 | 21,393 | 7,270 | 6,640 | 30,499 |
| ***Length of injecting career*** |  |  |  |  |  |  |  |  |  |  |
| Point estimate | 8,532 | 43,137 | 9,108 | 20 | 28 | 3,722 | 19,448 | 7,270 | 5,971 | 27,794 |
| Length of injecting career decreased by 25% | 4,838 | 40,700 | 9,093 | 12 | 27 | 3,705 | 19,478 | 7,158 | 6,005 | 27,820 |
| Length of injecting career increased by 25% | 11,129 | 44,819 | 9,120 | 26 | 28 | 3,733 | 19,427 | 7,357 | 5,948 | 27,726 |
| ***Force of infection*** |  |  |  |  |  |  |  |  |  |  |
| Point estimate | 8,532 | 43,137 | 9,108 | 20 | 28 | 3,722 | 19,448 | 7,270 | 5,971 | 27,794 |
| Force of infection decreased by 25% | 1,117 | 33,915 | 9,043 | 2 | 26 | 3,642 | 19,390 | 6,584 | 6,069 | 27,639 |
| Force of infection increased by 25% | 25,564 | 55,343 | 9,173 | 61 | 30 | 3,795 | 19,509 | 8,114 | 5,873 | 26,426 |
| ***PWID recruitment rate*** |  |  |  |  |  |  |  |  |  |  |
| Point estimate | 8,532 | 43,137 | 9,108 | 20 | 28 | 3,722 | 19,448 | 7,270 | 5,971 | 27,794 |
| PWID recruitment rate increased by 25% | 9,424 | 44,852 | 9,109 | 23 | 28 | 3,718 | 19,453 | 7,038 | 5,983 | 28,027 |
| PWID recruitment rate decreased by 25% | 7,940 | 41,965 | 9,110 | 18 | 28 | 3,724 | 19,446 | 7,432 | 5,964 | 27,644 |
| ***Discounting*** |  |  |  |  |  |  |  |  |  |  |
| Point estimate (3.5% discounting) | 8,532 | 43,137 | 9,108 | 20 | 28 | 3,722 | 19,448 | 7,270 | 5,971 | 27,794 |
| Discounting 0% | 8,532 | 43,137 | 9,108 | 20 | 28 | 3,917 | 21,476 | 5,342 | 7,646 | 51,663 |
| Discounting 7% | 8,532 | 43,137 | 9,108 | 20 | 28 | 3,579 | 18,018 | 9,109 | 4,735 | 16,204 |
| ***Testing costs*** |  |  |  |  |  |  |  |  |  |  |
| Point estimate | 8,532 | 43,137 | 9,108 | 20 | 28 | 3,722 | 19,448 | 7,270 | 5,971 | 27,794 |
| Testing costs halved (Ab and RNA) | 8,532 | 43,137 | 9,108 | 20 | 28 | 3,603 | 19,448 | 7,024 | 5,996 | 27,766 |
| Testing costs doubled (Ab and RNA) | 8,532 | 43,137 | 9,108 | 20 | 28 | 3,958 | 19,448 | 7,763 | 5,923 | 27,851 |
| ***Disease costs*** |  |  |  |  |  |  |  |  |  |  |
| Point estimate | 8,532 | 43,137 | 9,108 | 20 | 28 | 3,722 | 19,448 | 7,270 | 5,971 | 27,794 |
| Disease management costs halved | 8,532 | 43,137 | 9,108 | 20 | 28 | 3,219 | 19,448 | 13,855 | 5,324 | 26,547 |
| Disease management costs doubled | 8,532 | 43,137 | 9,108 | 20 | 28 | 4,224 | 19,448 | 686 | 6,619 | 29,041 |
| ***Health utilities when infected with hepatitis C*** |  |  |  |  |  |  |  |  |  |  |
| Point estimate | 8,532 | 43,137 | 9,108 | 20 | 28 | 3,722 | 19,448 | 7,270 | 5,971 | 27,794 |
| Health utilities are at their lower bound estimates | 8,532 | 43,137 | 9,108 | 20 | 28 | 3,722 | 19,448 | 5,207 | 5,971 | 27,794 |
| Health utilities are at their upper bound estimates | 8,532 | 43,137 | 9,108 | 20 | 28 | 3,722 | 19,448 | 12,268 | 5,971 | 27,794 |

Figure S1: Sensitivity analysis for incremental cost-effectiveness ratio (ICER; cost per quality-adjusted life year gained) of status-quo scenario versus no treatment scale-up at 2030.

Figure S2: Sensitivity analysis for net economic benefit at 2030 of status-quo scenario versus no treatment scale-up.

# Appendix B: model description

**A: Model description**

We used an open deterministic compartmental model of hepatitis C transmission, liver disease progression and the cascade of care based on Scott et al.^2,16^ (Figure S3).

*Populations*

The model considers three population groups: people who inject drugs (PWIDs), former PWIDs and the remaining general population. PWIDs could become former PWIDs due to cessation of injecting, and people from the general population can be recruited to become PWID.

*Infection status*

Individuals were classified as either susceptible (infection naïve or previously achieving spontaneous clearance or sustained viral response through treatment), acutely infected or chronically infected.

*Transmission*

For this study, infections were only modelled among the PWID population group (see calibration section for more), with susceptible PWID becoming infected at a rate proportional to the time-varying hepatitis C prevalence among PWID. Following infection, people were modelled to experience a short duration of acute infection, after which a proportion spontaneously cleared the virus and became susceptible again without treatment, and the remaining proportion became chronically infected.

*Liver disease*

METAVIR scores were used to classify everyone in the model according to stages of liver disease: F0, F1, F2, F3, F4, decompensated cirrhosis (DC), hepatocellular carcinoma (HCC) or post liver transplant. Liver disease progression was only modelled to occur for chronically infected individuals, with the exception of susceptible individuals with compensated cirrhosis (F4) who could develop DC or HCC.

*Care cascade*

Individuals who were chronically infected were also classified according to their care cascade status: infected and undiagnosed, infected and diagnosed antibody positive, infected and diagnosed RNA-positive, on treatment, or failed treatment.

*Testing and treatment*

In order for infected people to become cured, they need to be antibody tested, RNA tested and treated. Following cure, people in the model retained their liver disease (i.e. regression of disease was not included), and reinfection was allowed to occur among PWID at the same rate as initial infection (although re-infected PWID required an RNA test for screening). The number of tests and treatment available in each scenario and user inputs for the projection period.

*Mortality*

All-cause mortality was modelled to occur for everyone in the model, and additional injecting-related mortality was included for PWID and additional liver-related mortality was included for people with DC or HCC.

*Calibration*

A Particle Swarm Optimization Algorithm^54^ was used to best fit multiple model parameters to multiple epidemiological data points. For each setting being considered, the model was calibrated to time series data on the prevalence of hepatitis C among PWID, the prevalence of hepatitis C among the general population, the annual number of hepatitis C-related deaths, the total number of people living with hepatitis C, the estimated incidence of hepatitis C, and the proportion of people living with hepatitis C who were diagnosed. This involves simultaneously varying parameters for: the force of infection among PWID (the force of infection was dynamic and dependent on prevalence, but a constant scalar factor was varied), the disease progression rates (F0🡪F1, F1🡪F2, F3🡪F4, F4🡪DC, F4🡪HCC, DC🡪HCC), the annual probability of dying from DC, the annual probability of dying from HCC, and the annual probability of being diagnosed.

*Definition of targets*

The diagnosis and treatment targets were defined in the model, respectively, as:

- [total people diagnosed + cumulative successful treatments by 2030] / [total people living with hepatitis C in 2015 + cumulative new and re infections – HCV-related deaths among people living with HCV]; and
- [total people commenced treatment + cumulative successful treatments by 2030] / [total people diagnosed + cumulative successful treatments by 2030].

The successful treatments were included in the formulas to avoid pathological cases, such as scenarios where everyone was cured except for one undiagnosed person, which could be counted as missing the targets (because 0% would be diagnosed).


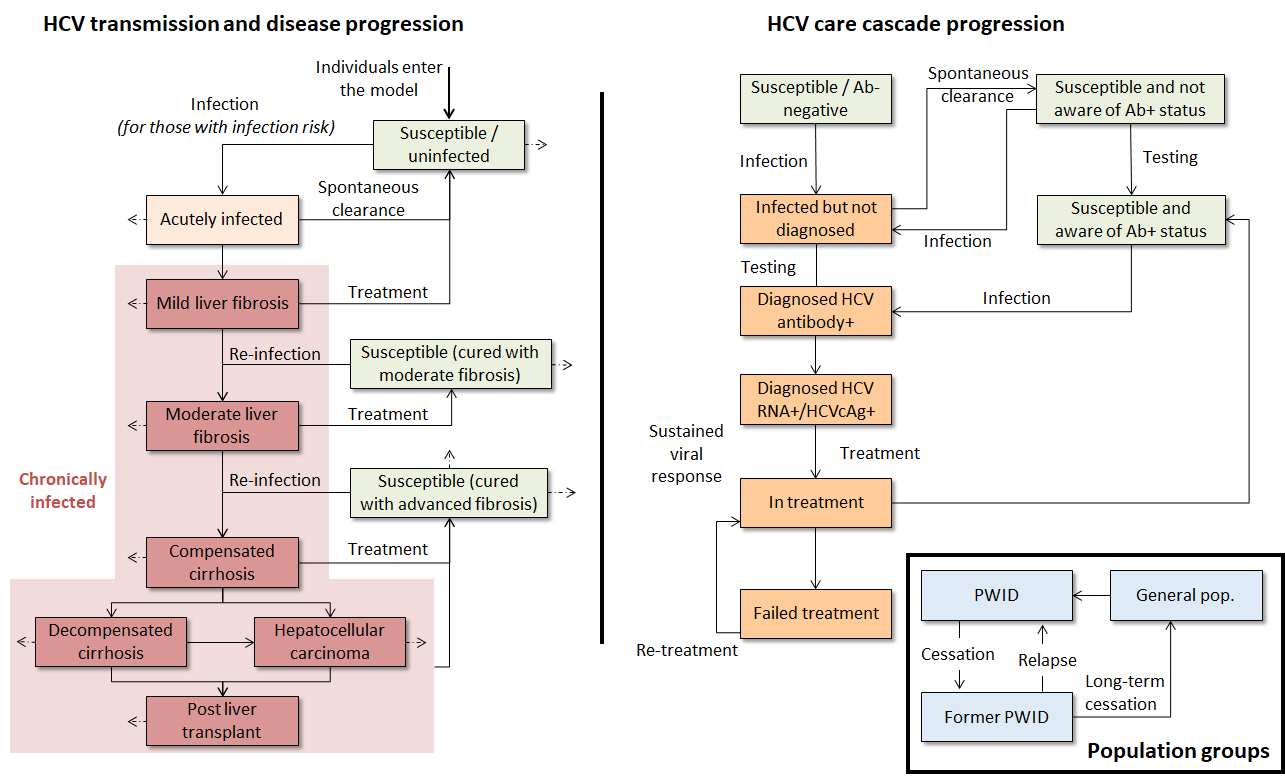


**Figure S3: HCV transmission, disease progression and care cascade model schematic.** Liver fibrosis stages F0, F1, F2, F3, F4 (compensated cirrhosis), decompensated cirrhosis and hepatocellular carcinoma were all explicitly modelled but are not shown for brevity.

*Capturing productivity losses*

The epidemiological model described above was used to capture direct costs (testing, treatment and disease management), but to estimate economic productivity losses an additional model was used. This model took as inputs the annual numbers people with hepatitis C and people who had been cured from hepatitis C, and produced the annual productivity losses from absenteeism and presenteeism (Figure S4). The calculation of productivity losses from premature deaths is described in the main manuscript.


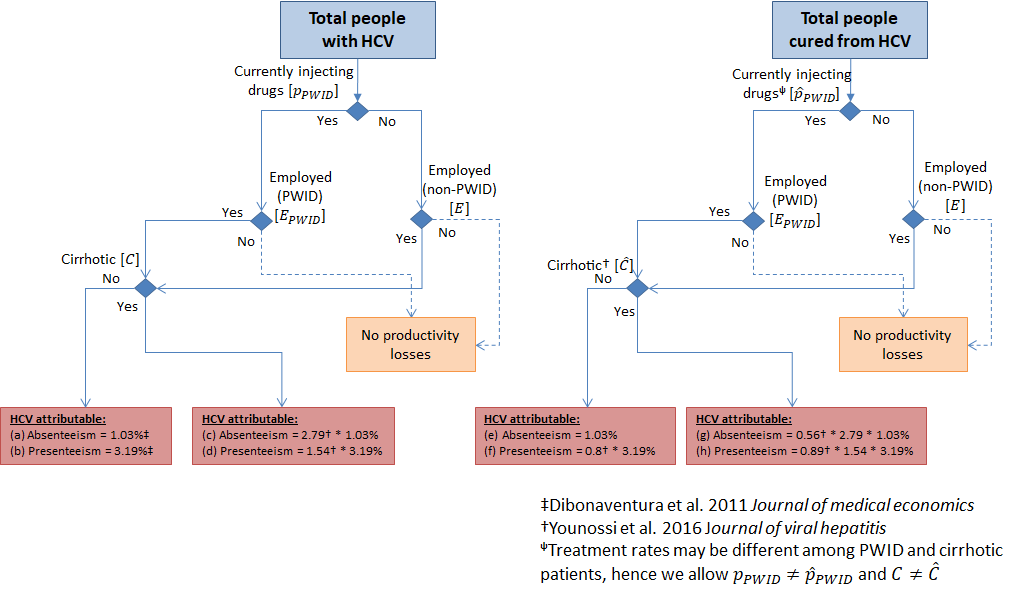


**Figure S4: Schematic of productivity model.**
